# Environmental Factors Variably Impact Tea Secondary Metabolites in the Context of Climate Change

**DOI:** 10.3389/fpls.2019.00939

**Published:** 2019-08-13

**Authors:** Selena Ahmed, Timothy S. Griffin, Debra Kraner, M. Katherine Schaffner, Deepak Sharma, Matthew Hazel, Alicia R. Leitch, Colin M. Orians, Wenyan Han, John Richard Stepp, Albert Robbat, Corene Matyas, Chunlin Long, Dayuan Xue, Robert F. Houser, Sean B. Cash

**Affiliations:** ^1^Food and Health Lab, Sustainable Food Systems Program, Department of Health and Human Development, Montana State University, Bozeman, MT, United States; ^2^Friedman School of Nutrition Science and Policy, Tufts University, Boston, MA, United States; ^3^Department of Biology, Tufts University, Medford, MA, United States; ^4^Tea Research Institute, Chinese Academy of Agricultural Sciences, Hangzhou, China; ^5^Department of Anthropology, University of Florida, Gainesville, FL, United States; ^6^Department of Chemistry, Tufts University, Medford, MA, United States; ^7^Department of Geography, University of Florida, Gainesville, FL, United States; ^8^Key Laboratory of Ethnomedicine of Ministry of Education, and College of Life and Environmental Sciences, Minzu University of China, Beijing, China; ^9^College of Life and Environmental Sciences, Minzu University of China, Beijing, China

**Keywords:** climate change, crop quality, secondary metabolites, food systems, agriculture

## Abstract

Climate change is impacting food and beverage crops around the world with implications for environmental and human well-being. While numerous studies have examined climate change effects on crop yields, relatively few studies have examined effects on crop quality (concentrations of nutrients, minerals, and secondary metabolites). This review article employs a culturally relevant beverage crop, tea (*Camelia sinensis*), as a lens to examine environmental effects linked to climate change on the directionality of crop quality. Our systematic review identified 86 articles as relevant to the review question. Findings provide evidence that shifts in seasonality, water stress, geography, light factors, altitude, herbivory and microbes, temperature, and soil factors that are linked to climate change can result in both increases and decreases up to 50% in secondary metabolites. A gap was found regarding evidence on the direct effects of carbon dioxide on tea quality, highlighting a critical research area for future study. While this systematic review provides evidence that multiple environmental parameters are impacting tea quality, the directionality and magnitude of these impacts is not clear with contradictory evidence between studies likely due to confounding factors including variation in tea variety, cultivar, specific environmental and agricultural management conditions, and differences in research methods. The environmental factors with the most consistent evidence in this systematic review were seasonality and water stress with 14 out of 18 studies (78%) demonstrating a decrease in concentrations of phenolic compounds or their bioactivity with a seasonal shift from the spring and /or first tea harvest to other seasons and seven out of 10 studies (70%) showing an increase in levels of phenolic compounds or their bioactivity with drought stress. Herbivory and soil fertility were two of the variables that showed the greatest contradictory evidence on tea quality. Both herbivory and soil fertility are variables which farmers have the greatest control over, pointing to the importance of agricultural management for climate mitigation and adaptation. The development of evidence-based management strategies and crop breeding programs for resilient cultivars are called for to mitigate climate impacts on crop quality and overall risk in agricultural and food systems.

## Introduction

### Climate Change and Crop Quality

Impacts of climate change on agricultural systems threaten crops with notable implications for farmer livelihoods and human health (Odada et al., 2008; Pachauri et al., 2014; Campbell et al., 2016). Since the 1950s, agricultural systems have experienced gradual systematic changes in average climate conditions including unprecedented multi-decadal warming, increased inter-annual variability of the Earth's surface temperatures, changes in average precipitation, greater weather variability, and more extreme weather conditions (Pachauri et al., 2014). These climatic changes have decreased agricultural productivity and shifted the geographic range of many crops (Lobell and Asner, 2003; Porter and Semenov, 2005). Concurrently, productivity of some crops has increased with a rise in temperatures (Ewert et al., 2005). Increasing atmospheric carbon dioxide (CO_2_) has decreases concentrations of micronutrients zinc (Zn) and iron (Fe) as well as protein for C_3_ grains and legumes (Myers et al., 2014). While the literature provides substantial evidence on the impact of climate change on crop yields (Lobell and Asner, 2003; Hertel et al., 2010; Lobell et al., 2011), fewer studies have focused on the effects of climate change on crop quality (Porter and Semenov, 2005; Ahmed and Stepp, 2016).

Crop quality is a multi-dimensional parameter that refers to the nutritional, health, and sensory attributes of crops as measured by the presence, absence, and/or concentrations of phytonutrients, minerals, and primary and secondary metabolites (i.e., bioactive food components or phytochemicals) as well as associated bioactivity, shelf life, and organoleptic properties (i.e., color, visual appeal, aroma, taste, and texture) (Mattos et al., 2014; Ahmed and Stepp, 2016). Secondary metabolites are non-nutrient plant constituents that impact crop quality through an influence on flavor, appearance, stability (Tomás-Barberán and Espin, 2001) and health-promoting attributes including mitigating micronutrient deficiencies (Johns and Sthapit, 2004) and the risk of diet-related chronic disease including cancer, heart disease, and diabetes (Liu, 2013). Over 100,000 secondary metabolites have been identified in five chemical classes including phenolics, terpenes, alkaloids, and other nitrogen-containing compounds, phytosterols, and organosulfur compounds (Goldberg, 2003; Swift et al., 2004).

Plants have evolved secondary metabolites to protect themselves from various abiotic and biotic stressors (Fraenkel, 1959; Coley et al., 1985). Since the synthesis of secondary metabolites represents a metabolic cost, plants tend to produce these compounds in notable concentrations if they have the ecological cue to do so based on interactions between environmental (Lower and Orians, 2003; Björkman et al., 2011; Kowalsick et al., 2014), agricultural (Ahmed et al., 2013), genetic (van-Dam and Vrieling, 1994), and physiological factors (Coley et al., 1985; Glynn et al., 2007). Each crop has specific secondary metabolites that characterize crop quality while the presence of other secondary metabolites is viewed as deleterious for crop quality due to off-flavors or toxicity attributes. Climate change factors have resulted in a decrease of multiple secondary metabolites in a range of food and beverage crops including coffee (Villarreal et al., 2009), tea (Ahmed et al., 2014a,b; Kowalsick et al., 2014), grapes (Xu et al., 2011), brown rice (Britz et al., 2007), kale (Zietz et al., 2010), tomatoes (Kacjan-Maršič et al., 2010), and peanuts (Casini et al., 2003).

### Tea as a Model System for Examining Climate Effects on Crop Quality

This review article employs tea (*Camelia sinensis*; Theaceae) to understand how climate change impacts crop quality of a culturally-relevant beverage plant. Tea, the botanical source of all white, green, oolong, black and pu-erh tea beverages, was selected as a study system because of its prevalence in diets globally, its production in over 50 countries on five continents (FAOSTAT, 2016), and because it is a plant that is cultivated for its quality based on secondary metabolites (Ahmed et al., 2010). Polyphenolic catechin, methylxanthine (i.e., caffeine), and volatile secondary metabolites, coupled with carbohydrates and amino acids, contribute to tea quality through influencing the flavor and appearance of tea (Drewnowski and Gomez-Carneros, 2000; Scharbert et al., 2004) as well as its health claims including anti-oxidative, anti-inflammatory, neuro-protective, cardio-protective, anti-cancer, anti-microbial, and anti-atherosclerotic activities (Trevisanato and Kim, 2000; Lin et al., 2003; Clement, 2009). Tea is further suitable as a study system to examine the effects of climate change because it is a woody perennial that experiences multiple decadal effects of climate change. Successful tea cultivation tea is dependent on climatic conditions including temperature, rainfall, humidity, and solar radiation (Wijeratne, 1996).

Marx et al. (2017) identified 14 articles highlighting that shifts in rainfall and solar radiation influence tea yields and quality with implications for farmer livelihoods. For example, Biggs et al. (2018) demonstrate that climate change is impacting farmer livelihoods across four major tea-growing regions of Assam, India. Boehm et al. (2016) show that a decrease in solar radiation in the previous growing season is associated with lower tea yields. However, the literature is missing a systematic review that synthesizes the evidence of environmental effects on tea quality and, the directionality of changes in quality parameters.

This study addresses the aforementioned knowledge gap through a systematic literature review that examines the following question: *What are effects of environmental variation related to climate change on tea quality?* Given the lack of studies examining long-term effects of climate change on tea quality, we synthesize studies that examine the effects of environmental factors that are shifting with climate change. This review can inform future research by identifying literature gaps and new research questions while serving as a model for literature reviews on climate change effects on crop quality.

## Systematic Review Methods

The Preferred Reporting Items for Systematic Reviews and Meta-Analyses (PRISMA; Moher et al., 2009) and the Guidelines for Systematic Review and Environmental Synthesis in Environmental Management (Collaboration for Environmental Evidence, 2013) were applied to design a systematic review protocol to collect evidence on the following closed-frame study question: *What are effects of environmental variation related to climate change on tea quality?* We used the PICO (Population, Intervention/Exposure, Comparator, Outcome) framework elements (Schardt et al., 2007) to structure our systematic review.

The biological population/study unit is tea (*Camellia sinensis* L.; Theaceae). The intervention/exposure that we examined is environmental variability linked to climate change, which includes variables including temperature, precipitation, elevation, seasonality, location, herbivory, soil, and solar radiation. As we found a lack of published studies directly examining climate effects on tea quality based on our preliminary review, we included environmental factors that vary with global change in systematic review search protocol. The comparator assessed in our review was variation in a specific environmental variable from a baseline evaluation or other comparison. The outcomes that we examined were impacts on the biochemical parameter of tea quality including secondary metabolite chemistry that consists of phenolic catechins, methylxanthines (including caffeine), amino acids (including theanine), and a range of volatile compounds.

A multidisciplinary team of subject experts and a review methodology expert collaboratively designed the systematic review protocol. Specifically, the multidisciplinary team of subject experts included scientists with expertise in the fields of plant biology, chemistry, soil science, herbivory, phytochemistry, biochemistry, agronomy, food science, tea science, and climate change. Establishment of the review protocol involved preliminary scoping of search terms to test the search strategy for indication of the volume of relevant literature. Search terms were identified and revised during an iterative process after examining relevant articles from the search and refining search terms to meet both evidence needs as well as feasibility for a systematic review. Following are the key components of the final search terms that included a combination of tea, environmental factor linked to climate change, and quality parameter: “tea OR camellia sinensis” AND “climate change OR global warming OR season OR solar radiation OR precipitation OR geographic area OR temperature OR soil OR wind OR annual bud burst OR carbon dioxide” AND “antioxidant OR caffeine OR phytochemical OR catechin OR theaflavin OR flavonol OR polyphenol OR secondary metabolite.” Supplementary Table 1 lists the final search terms used in this systematic review. The final search terms were tested with a set of known relevant articles.

A total of five publication databases were searched: PubMed, Web of Science, EBSCO GreenFILE, Scopus, and, Agricola. Databases were selected that are commonly used to search for articles in the areas of food systems, agriculture, climate change, secondary metabolite chemistry, and human health. Inclusion criteria were restricted to peer-review articles published in the English language from 2000 to 2015. The software program Covidence was used to manage articles identified within the publication databases and was further used for screening of articles. Following the search, the retrieved articles were screened by a review panel of three reviewers (KS, MH, and SA) to minimize reviewer bias in identifying articles for their relevance to the review question using *a-priori* inclusion criteria. Two reviewers from the review panel screened each article. Decision discrepancies (inclusion or exclusion) were discussed by the entire panel for resolution. The final set of articles was critically appraised for their design on focusing on environmental effects on tea quality.

The final set of articles in the study was read by a separate data extraction review panel (DK, DS, and AL). Appropriate data from each of these articles were extracted by one of three members of the data extraction panel and placed into a table highlighting specific study parameters including environmental factor, variety and cultivar of tea, quality indicator, method(s) of analysis, and outcomes. A second member on the data extraction panel reviewed extracted data in the table to verify that appropriate data was extracted.

The final search resulted in the retrieval of 9,750 articles including 1,754 duplicates for a total of 7,996 articles (Supplementary Figure 1). From this, 93 articles were identified by the review panel for inclusion in the review. These articles were relevant to the review question using our *a priori* inclusion criteria. Nine of these articles were removed from the search because it was not possible to retrieve the full text of these papers, resulting in a total of 84 articles that were included in the synthesis of our systematic review.

Each article included in the study was grouped on the basis of which environmental factor(s) were assessed in the study, and then placed in a corresponding table of the most prevalent environmental factors that emerged from the systematic review. Articles were placed in multiple tables if they examine multiple environmental variables. For each environmental factor table, we applied a coding framework to quantify the number of articles that demonstrated directional changes (increase, decrease, or no change) of specific tea quality parameters. Specifically, we quantified the following quality parameters: (1) increase, decrease, or no change in catechins and other phenolic compounds with an increase in [environmental factor, i.e., temperature], (2) increase, decrease, or no change in catechins and other phenolic compounds with a decrease in [environmental factor, i.e., temperature], (3) increase, decrease, or no change in caffeine with an increase in [environmental factor, i.e., temperature], (4) increase, decrease, or no change in caffeine with a decrease in [environmental factor, i.e., temperature], (5) increase, decrease, or no change in terpenoids and other volatiles with an increase in [environmental factor, i.e., temperature], (6) increase, decrease, or no change in terpenoids and other volatiles with a decrease in [environmental factor, i.e., temperature], (7) increase, decrease, or no change in amino acids with an increase in [environmental factor, i.e., temperature] and, (8) increase, decrease, or no change in amino acids with a decrease in [environmental factor, i.e., temperature]. Unfortunately, as the studies used different measures of crop quality and variable experimental designs, we were unable to quantitatively compare between studies. Three reviewers (DS, DK, and SA) coded the outcomes for each article to ensure there were no discrepancies. The resulting data were summarized into a narrative synthesis and a quantitative synthesis.

Some environmental factors were merged together due to their relatedness and presentation in the literature. Geographic area was separated from altitude as it was presented separately in the literature. While the systematic review did not identify a study on carbon dioxide effects on tea quality, we summarize a recent study outside of the time frame as this is a critical abiotic stressor associated with climate change. Those studies that included multiple variables were analyzed individually for all relevant environmental factors.

## Results

Findings from the systematic review highlighted the following environmental factors as being prevalent in the literature as impacting tea quality:

1. seasonality (including change of harvest season such as shifts between spring, summer, autumn, and winter harvest seasons or shifts between dry and wet seasons as well as bud burst; 18 studies; Supplementary Table 2);
2. water stress (including precipitation; 10 studies; Supplementary Table 3);
3. geography (8 studies; Supplementary Table 4);
4. light factors (including solar radiation; 6 studies; Supplementary Table 5);
5. altitude (5 studies; Supplementary Table 6);
6. herbivory and microbes (5 studies; Supplementary Table 7);
7. temperature (4 studies; Supplementary Table 8) and;
8. soil and nutrient factors (27 studies; Supplementary Table 9).

The most prevalent crop quality parameters in the literature addressing the study question were total phenolic concentration, catechin content [including epigallocatechin gallate (EGCG), epicatechin gallate (ECG), epigallocatechin (EGC), and catechin gallate (CG)], and antioxidant activity. Other prevalent quality parameters include caffeine, theaflavin, thearubigin, tannins, carotenoids, terpene alcohols (i.e., linalool and geraniol) and other aromatic volatiles, fatty acid composition, chlorophyll, glycoside precursors, proanthocyanidin, and lipoxygenase and glycosidase enzymatic activities. Overall, increases in the aforementioned secondary metabolites is associated with increased crop quality until a specific threshold for human flavor preference when a further increase is no longer considered desirable. Total color, brightness, and flavor of brewed tea were measured as organoleptic quality parameters linked to secondary metabolite concentrations. High-performance liquid chromatography (HPLC) and colorimetric spectrophotometry assays were the most prevalent methods for analyzing tea quality.

**Figure 1:**
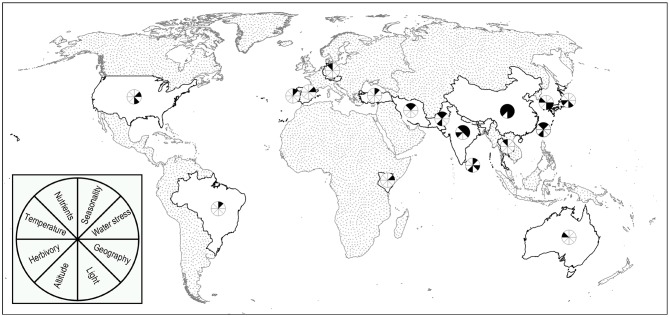
Environmental factors assessed by location. Map depicting the range of tea producing and consuming countries where the studies identified in this systematic review were carried out along with the corresponding environmental factors assessed.

The reviewed studies were carried out in a range of tea producing and consuming countries (Figure 1). In addition, the studies included a wide range of different tea cultivars of the *assamica* and *sinensis* varieties of *Camellia sinensis* including AC-259, T-78, B-157, B-777, S-449, UPASI-9, UP-2, UP-3, UP-8, BSS-2, TV-23, TV-25, TV-26, ZC108, ZC302, LJ 43, Derepazari 7, Fener, Yulan, and Fudingdaba. However, many of studies did not specify the variety, cultivar, or landrace of the tea examined. The lack of information on the variety of tea as well as the broad range of cultivars examined prevented analysis of the response of tea plants to environmental variables on the basis of tea variety and cultivar.

### Seasonality

**Figure 2:**
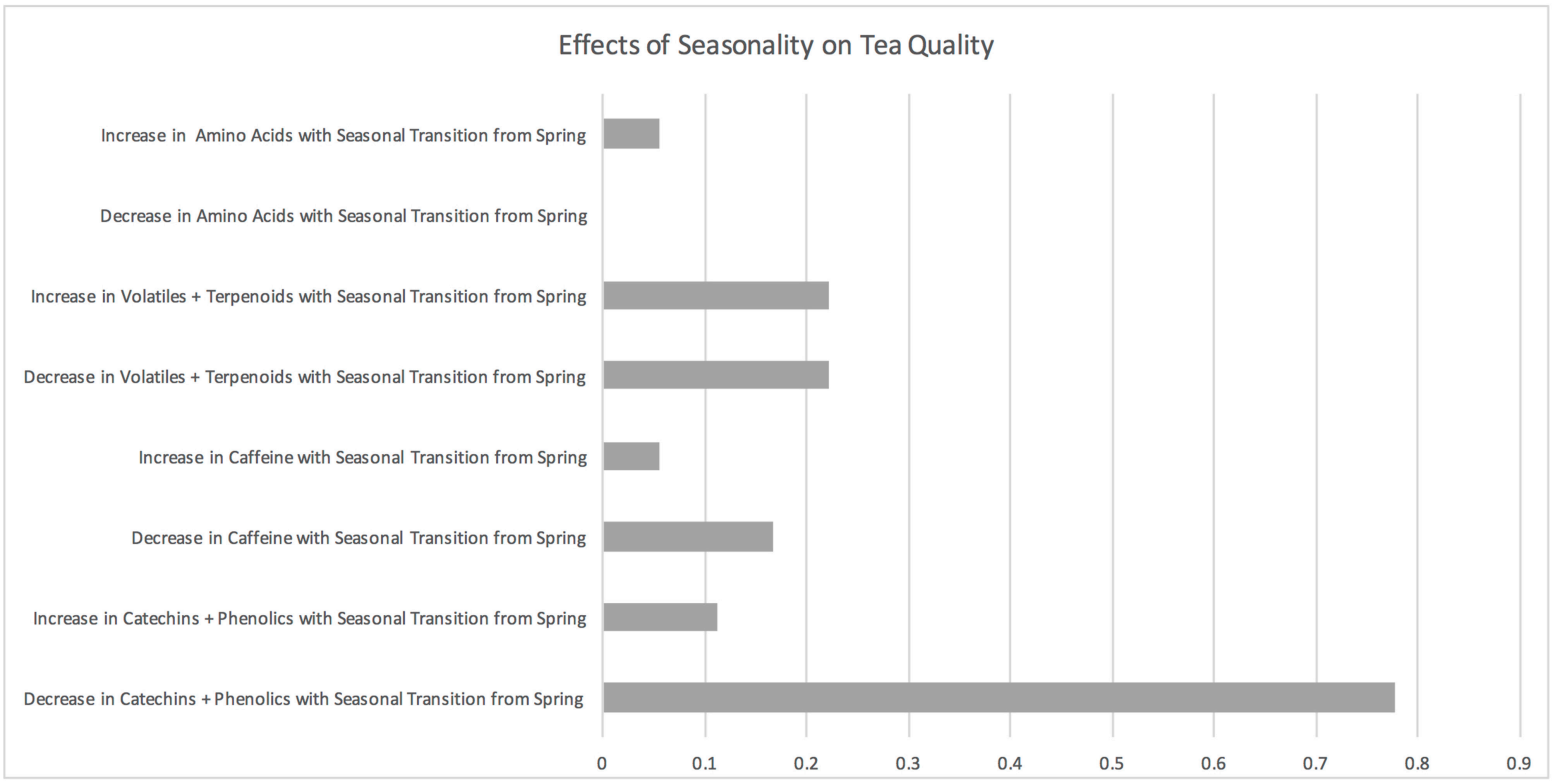
Effects of seasonality on tea quality. The horizontal axis depicts the percentage of studies and the vertical axis depicts directional changes in key quality parameters corresponding to climate change related environmental shifts.

One-fifth (18 of 86) of the studies reviewed assessed the effects of seasonality factors on tea quality including change of harvest season between spring, summer, autumn, and winter harvest seasons or shifts between dry and wet seasons as well as bud burst (Figure 2; Supplementary Table 2). The main seasonality factor examined was comparing the spring harvest to either the summer, monsoon, autumn, and winter, with a comparison between the spring and monsoon season being the most prevalent seasonal comparator. The spring tea harvest season is generally the first harvest season that follows winter dormancy in direct response to increased temperatures (Ahmed and Stepp, 2012). The main quality parameters measured in reviewed studies on seasonality were phenolic secondary metabolites (including individual catechin compounds) and / or antioxidant activity (14 studies), followed by volatile metabolites (4 studies), and caffeine (a methylxanthine compound; 4 studies).

Fourteen seasonality studies demonstrated that concentrations of individual catechins, phenolic secondary metabolites, and / or antioxidant activity decreased with a seasonal shift from the spring and/or first tea harvest to other seasons, particularly the monsoon season (Sud and Baru, 2000; Saikia and Mahanta, 2002; Akhlas, 2003; Venkatesan et al., 2007; Chen et al., 2010, 2014; Kottur et al., 2010; Ansari et al., 2011; Jayasekera et al., 2011, 2014; Xu et al., 2012; Ahmed et al., 2014b; Baptista et al., 2014; Topuz et al., 2014). Phenolic catechin compounds are generally associated with high tea quality; however, relatively high concentrations of particular phenolics are linked to a bitter or astringent taste that is generally less desired by consumers (Ahmed et al., 2014b). Two seasonal studies showed that while some phenolic secondary metabolites, and/or antioxidant activity decreased with a seasonal shift from the spring and / or first tea harvest to other seasons, other secondary metabolites and / or antioxidant activity increased. Ahmed et al. (2014b) demonstrated that concentrations of desirable phenolic catechins were up to 50% lower during the beginning of the monsoon season compared to the spring, while total phenolic concentrations and antioxidant activity increased.

The four seasonality studies measuring tea volatiles demonstrated that some volatile compounds increased with the seasonal transition from the spring and/or first tea harvest and that other compounds decreased (Saikia and Mahanta, 2002; Rawat and Gulati, 2008; Tontul et al., 2013; Kowalsick et al., 2014). Kowalsick et al. (2014) demonstrated that the number and concentration of over 300 volatile compounds from 18 chemical families either increased, decreased, or stayed the same with the onset of the monsoon from the spring season. This study showed that in general the spring season has a greater number and higher concentration of volatiles associated with high tea quality (Kowalsick et al., 2014). Three studies showed that caffeine concentrations decreased with a transition from the spring season to the summer monsoon season (Akhlas, 2003; Ansari et al., 2011; Ahmed et al., 2014b) and one study found caffeine to increase during the summer (Saito et al., 2007). One study provided evidence of an increase in amino acids with the transition from the spring to the summer monsoon (Saikia and Mahanta, 2002).

Six of the 14 seasonality studies provided evidence that while some quality parameters decreased with a shift in season, other measures of quality increased (Saikia and Mahanta, 2002; Venkatesan et al., 2007; Rawat and Gulati, 2008; Tontul et al., 2013; Ahmed et al., 2014b; Kowalsick et al., 2014). Rawat and Gulati (2008) found that total content of glycosidic bound flavor compounds was highest during early growth flush (April—mid June), declined during rains (mid June—September), and exhibited recovery during the backend flush (mid September—November). The authors further found an increase in the terpene index and a decrease in terpenoid/non-terpenoid ratio during the rains due to a marked decrease in terpenoid compounds (mainly aromatic geraniol). Jayasekera et al. (2011) found that variation of tea quality on the basis of season was further dependent on geographic location with some plantations having higher quality during the dry season and others having higher quality during the monsoon.

### Water Stress

**Figure 3:**
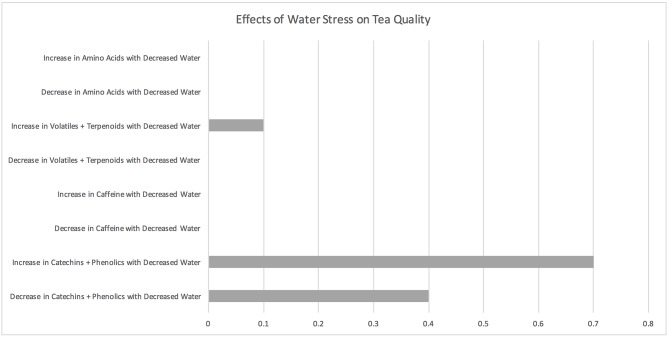
Effects of water stress on tea quality. The horizontal axis depicts the percentage of studies and the vertical axis depicts directional changes in key quality parameters corresponding to climate change related environmental shifts.

A total of 10 studies examined responses of tea plants to low water availability, including drought stress and soil moisture content (Figure 3; Supplementary Table 3). Five of these studies showed an increase in phenolic compounds and/or antioxidant activity with drought stress (Hernández et al., 2006; Upadhyaya et al., 2011, 2013; Ahmed et al., 2013; Bhattacharya et al., 2014) while two studies showed a decrease in these compounds (Cheruiyot et al., 2007, 2008) and two other studies showed both an increase and decrease in these compounds with drought stress (Chakraborty et al., 2002; Upadhyaya and Panda, 2004). Hernández et al. (2006) found that phenolics (including epicatechin and epigallocatechin gallate) accumulated in drought-stressed tea plants. Cheruiyot et al. (2007) found that declining soil water content over a 12-week period significantly reduced growth and total polyphenolic concentrations in tea shoots depending on cultivar type. Chakraborty et al. (2002) highlight that while phenolic secondary metabolites initially increase with drought stress, they decrease during extended drought stress.

One water stress study examined the effect of drought stress on levels of volatile compounds (Cao et al., 2007). Cao et al. (2007) showed that the number of aromatic constituents in fresh tea leaves was highest under a soil water content of 54% (relative to field capacity) and lowest under a soil relative water content of 100%. None of the water stress studies examined the effects of water stress on caffeine levels in tea.

### Geography

**Figure 4:**
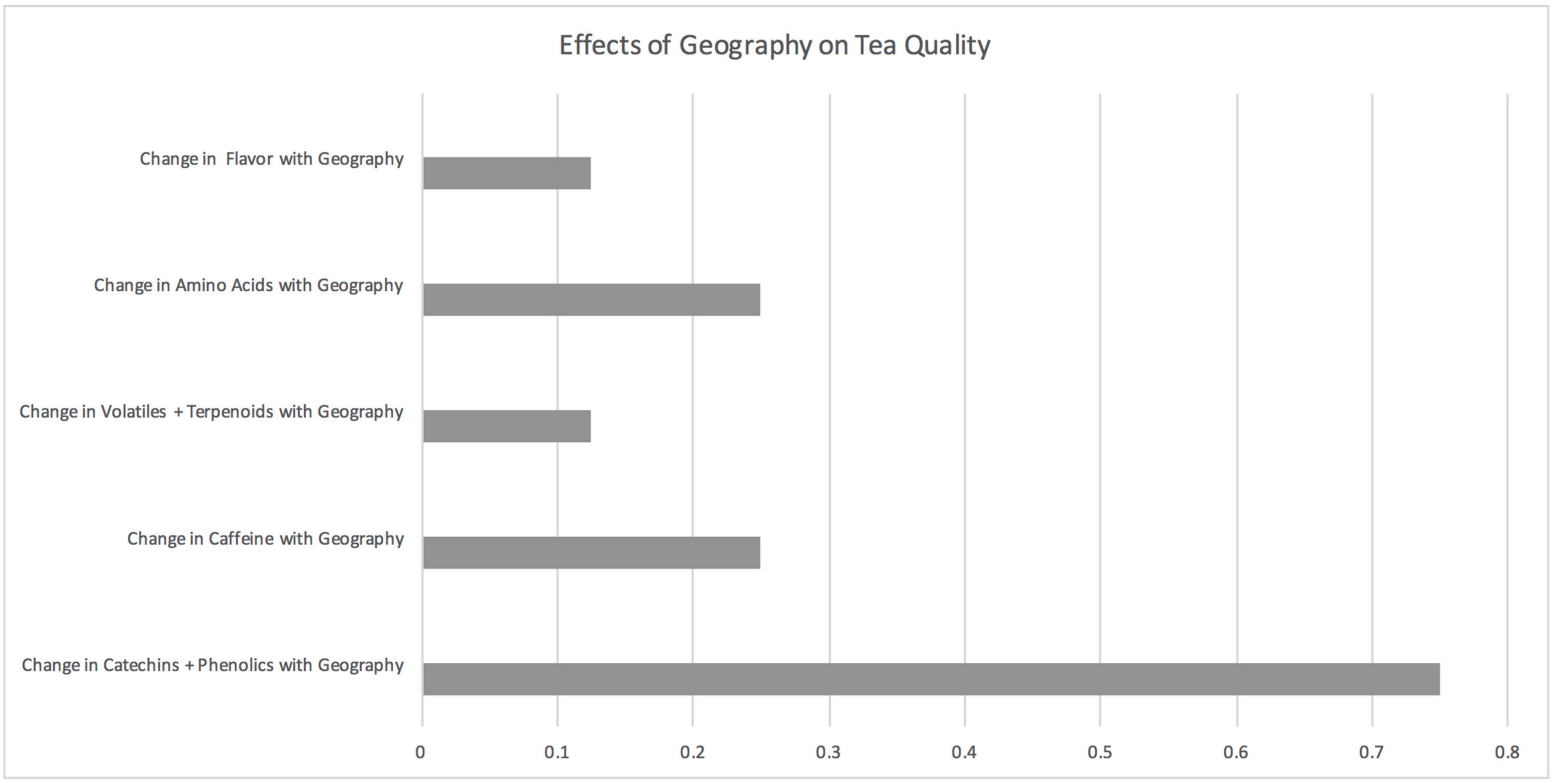
Effects of geography on tea quality. The horizontal axis depicts the percentage of studies and the vertical axis depicts directional changes in key quality parameters corresponding to climate change related environmental shifts.

While geography is not directly changing with climate change, areas that are suitable for agriculture are shifting with climate change. The eight studies examining the effects of geography on tea quality (Figure 4; Supplementary Table 4) focused on agroclimatic zones, location, and karst topography. Geography was found to notably impact catechins and other tea polyphenols in seven studies (Borse et al., 2002; Li et al., 2007; Bhuyan et al., 2009, 2013; Lee et al., 2015). Two studies showed that caffeine varied with geography (Borse et al., 2002; Lee et al., 2010), two studies showed that amino acids varied with geography (Lee et al., 2010, 2015), and no studies showed that volatiles varied with geography. One study found that location notably impacts sensory parameters of tea and that this directly corresponds to higher phenolic theaflavin and therubigin tea constituents (Bhuyan et al., 2009).

### Light Factors

**Figure 5:**
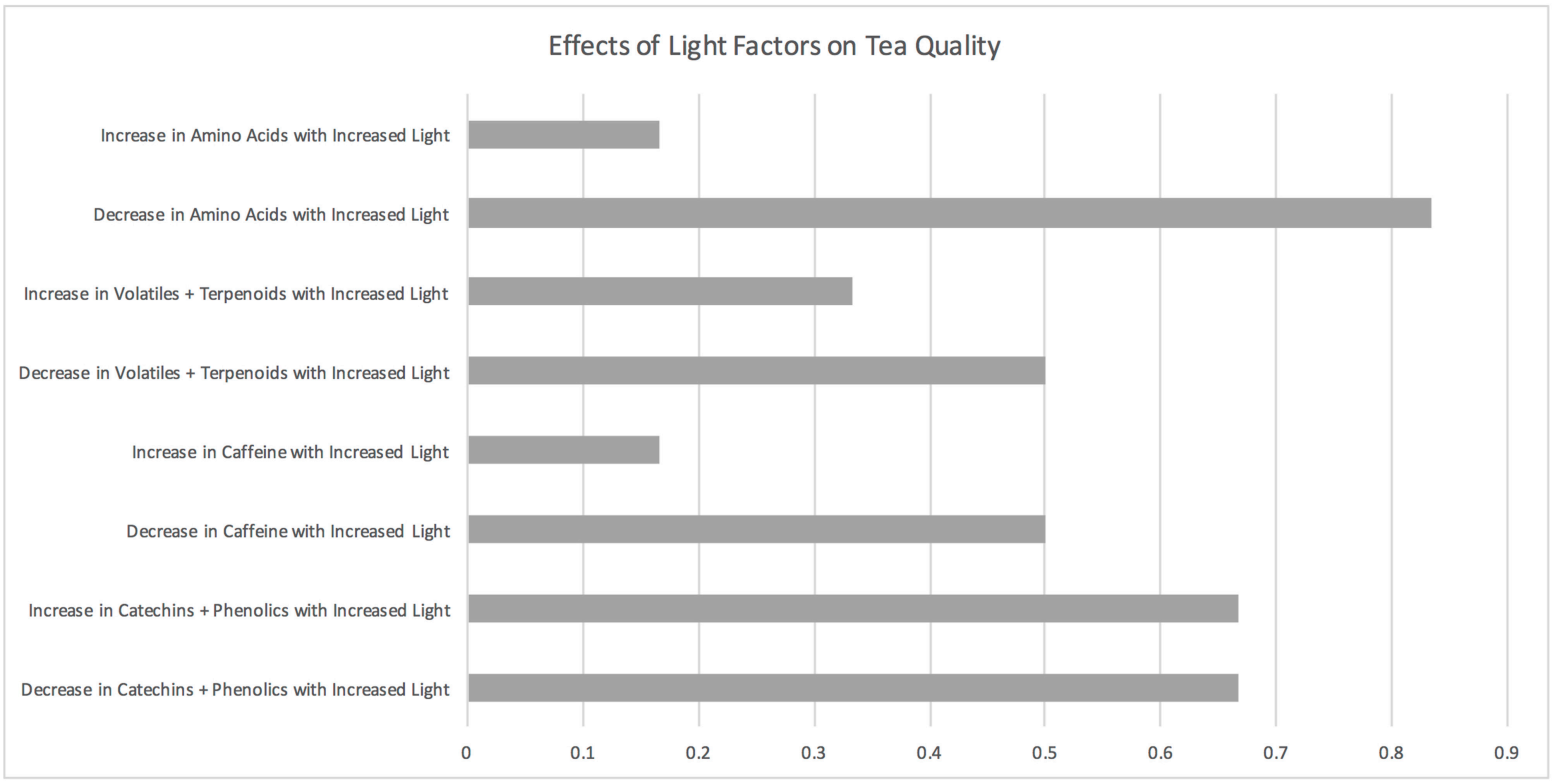
Effects of light factors on tea quality. The horizontal axis depicts the percentage of studies and the vertical axis depicts directional changes in key quality parameters corresponding to climate change related environmental shifts.

The six studies on the effects of light factors explored shading treatment, light intensity, and light quality (Figure 5; Supplementary Table 5). One of these studies found that polyphenols increased with an increase in light (Zhang et al., 2014) while one study found an inverse relationship of light and polyphenols (Zheng et al., 2008) and two studies showed that some polyphenols increase with an increase in light and others decrease (Ku et al., 2010; Song et al., 2012). Ku et al. (2010) found that shade cultured green tea (tencha) had lower levels of phenolic epigallocatechin and epicatechin compounds as well as higher levels of amino acids compared to unshaded green tea while having higher levels of gallocatechin. These difference in polyphenol profiles resulted in the shade cultured tea to have higher umami profile and less astringent taste than the unshaded tea.

Three light studies found that caffeine levels decrease with an increase in light (Song et al., 2012; Lee et al., 2013; Zhang et al., 2014) while one of these studies found that caffeine levels increased at specific amounts of light (Song et al., 2012). Song et al. (2012) demonstrated a non-linear relationship between shade levels (10% shade to 60% shade) and tea quality based on six secondary metabolites (L-theanine, caffeine, -epicatechin, -epicatechin gallate, -epigallocatechin gallate, and –epigallocatechin). Three of the light studies indicate that some terpenoids and other volatiles increase while others decrease with a change in light levels (Ku et al., 2010; Song et al., 2012; Zhang et al., 2014). Overall, increased light results in decreased concentrations of amino acids (Ku et al., 2010; Song et al., 2012; Deng et al., 2013; Lee et al., 2013; Zhang et al., 2014).

### Altitude

**Figure 6:**
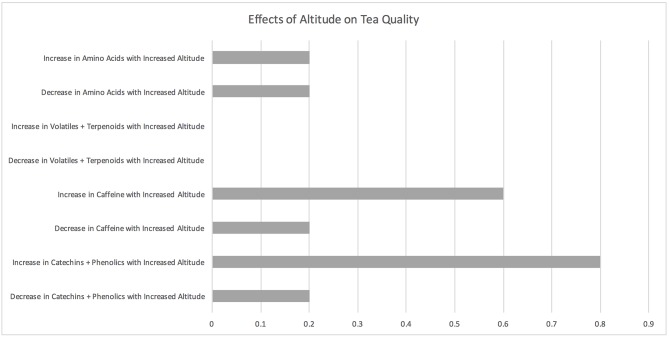
Effects of altitude on tea quality. The horizontal axis depicts the percentage of studies and the vertical axis depicts directional changes in key quality parameters corresponding to climate change related environmental shifts.

Studies demonstrate that higher elevation is generally associated with higher tea quality as measured by higher levels of catechins and other polyphenols as well as caffeine (Figure 6; Supplementary Table 6). Four studies found that tea catechins and other polyphenols increased with elevation (associated with cooler temperatures and higher oxidative stress) (Akhlas, 2003; Abeywickrama et al., 2010, 2011; Ohno et al., 2011) and one study found lower catechin levels with higher elevation (Chen et al., 2014). Three studies found that caffeine was higher in tea cultivated at higher elevation as compared to lower elevation (Akhlas, 2003; Abeywickrama et al., 2010; Ohno et al., 2011) and one study found lower caffeine levels with higher elevation (Abeywickrama et al., 2011). One study demonstrated that amino acid levels increase with higher elevation compared to lower elevation (Ohno et al., 2011) while one study found that amino acids were higher for lower elevation tea samples (Abeywickrama et al., 2011). One recent study (not included in this review) demonstrated an increase in favorable aromatic compounds that have sweet, floral, and honey-like notes with an increase in elevation while exhibiting a decrease in caffeine, epicatechin gallate, gallocatechin, and catechin (Kfoury et al., 2018).

### Herbivory and Microbes

**Figure 7:**
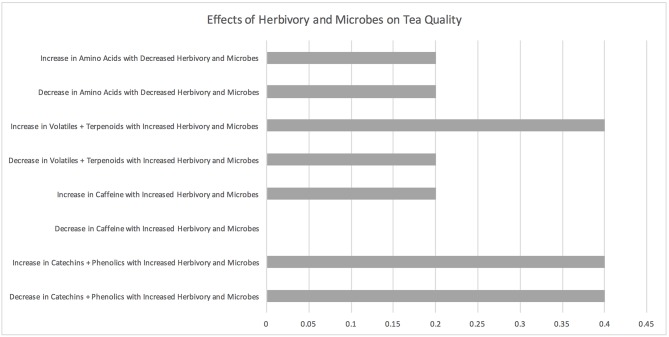
Effects of herbivory on tea quality. The horizontal axis depicts the percentage of studies and the vertical axis depicts directional changes in key quality parameters corresponding to climate change related environmental shifts.

Three studies on herbivory and two studies on microbes examined the effects of various insects (tea aphids, tea mosquito bug, and Kanzawa spider mites) and microbes (*phomospsis* and *Arbusular mucro* fungi from natural and cultivated tea rhizospheres) (Figure 7; Supplementary Table 7). Herbivory resulted in either a decrease in catechins (1 study; Chakraborty and Chakraborty, 2005), increase in catechins (1 study; Dong et al., 2011), increases in volatiles (1 study; Dong et al., 2011), and increase in some volatiles and decrease in other volatiles (1 study; Han and Chen, 2002). Han and Chen (2002) found that tea aphid-damaged tea shoots showed the presence of volatile compounds benzaldehyde and E-2-hexenoic acid which favorably contribute to aroma. Intact tea shoots on the other hand had butanoic acid-3-hexenyl ester and 1-octanol. None of the herbivory studies demonstrated that caffeine and amino acid levels were impacted.

One microbe study focused on the pathogen *phomospsis* (Ponmurugan and Baby, 2007) and the other focused on the healthy soil fungi *Arbusular mucro* (Singh et al., 2010). The presence of the pathogen reduced polyphenol and amino acid levels in Ponmurugan and Baby (2007). Inoculating tea plants with arbuscular mycorrhizal fungi increased catechin and other polyphenol levels in tea as well as amino acids and caffeine (Singh et al., 2010).

### Temperature

**Figure 8:**
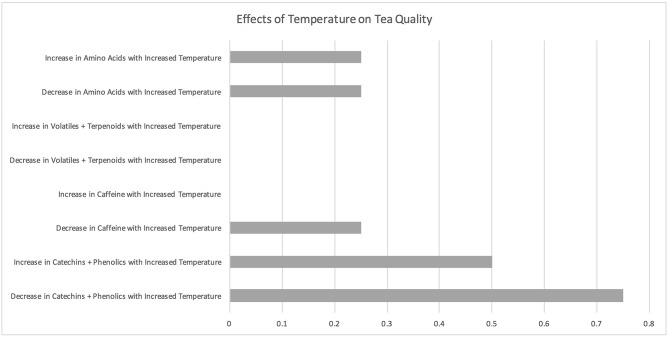
Effects of temperature on tea quality. The horizontal axis depicts the percentage of studies and the vertical axis depicts directional changes in key quality parameters corresponding to climate change related environmental shifts.

Three of the four temperature studies (Figure 8; Supplementary Table 8) found an inverse relationship between temperature and tea quality on the basis of catechins, phenolic secondary metabolites, and antioxidant compounds/activity (Lee et al., 2010; Wang et al., 2011; Wei et al., 2011) while one study showed that increased temperature resulted in increased catechin compounds (Yao et al., 2005). Wei et al. (2011) showed that higher catechin content was related to lower temperatures (same as higher elevation). Yao et al. (2005) showed that tea quality as determined by key catechin compounds [(-)-epigallocatechin gallate, (-)-epicatechin gallate, and catechin gallate] was higher during warm months and lower during cool months. Wang et al. (2011) showed that increased temperatures were associated with an increase in (-)-epigallocatechin, (-)-epicatechin, (-)-epicatechin gallate, and (-)-epigallocatechin gallate while catechin (C) decreased. Only one temperature study examined caffeine levels in tea (Lee et al., 2010). Lee et al. (2010) showed that green tea samples grown at high temperature had lower levels of caffeine and catechin compounds compared to those at lower temperature.

### Soil and Nutrient Factors

The 26 soil studies examined a range of soil nutrients including aluminum, zinc, iron, nitrogen, phosphorus, magnesium, selenium, manganese, potassium, and boron (Al, Zn, Fe, N, P, Mg, Se, Mn, K, and B, respectively) as well as pH levels (Supplementary Table 9). Broadly, these studies have two (sometimes overlapping) foci: (1) micro-nutrient fertility, and (2) macro-nutrient fertility, with several of the micro-nutrient studies assessing toxicity. Several of the reviewed soil studies assessed the impacts of both deficiency and sufficiency on quality parameters. These studies highlight how soil and nutrient factors notably impact catechin and other phenolic compounds (Hu et al., 2003; Ruan et al., 2007a; 2010, 2013; Chen et al., 2011; Jayaganesh et al., 2011; Duan et al., 2012; Lin et al., 2012; Sae-Lee et al., 2012; Yang et al., 2014), antioxidant defense enzymes (Hajiboland et al., 2013), caffeine (Yang et al., 2014; Sedaghathoor et al., 2019), amino acids (Hu et al., 2003; Venkatesan et al., 2004, 2005; Ruan et al., 2007a; 2010, 2013; Jayaganesh and Venkatesan, 2010; Jayaganesh et al., 2011; Lin et al., 2012; Yang et al., 2014), vitamins (Hu et al., 2003), and flavor (Venkatesan et al., 2006). Each of these is summarized below (Supplementary Table 9).

Although many crop species are inhibited by high levels of soluble aluminum (Al) in the soil, most often found at low soil pH (e.g., pH < 5.0), we found multiple studies that demonstrated that Al stimulates tea growth (Mukhopadyay et al., 2012), phytochemical concentration associated with high-quality tea (Chen et al., 2011; Duan et al., 2012; Sae-Lee et al., 2012; Hajiboland et al., 2013), or both. Both Duan et al. (2012) and Sae-Lee et al. (2012)found that increasing Al resulted in higher polyphenol, caffeine, and amino acid content of tea leaves; Sae-Lee et al. (2012) also noted higher content of catechins with increasing Al, and also with increasing Se. Hajiboland et al. (2011) found that Al supplementation could compensate for B deficiency.

The stimulation of tea growth resulted in increased amino acid and vitamin C from application of Se (Hu et al., 2003). Deficiency and toxicity impacts from Zn (Mukhopadhyay et al., 2013) and Fe (Hemalatha and Venkatesan, 2011) were observed on growth and enzymatic activity in tea; Venkatesan et al. (2007) also observed that excess Mn resulted in lower amino acid and carotenoid content of tea leaves. Three papers evaluated the impacts of Mg fertilization on growth and composition of tea. The work of Ruan et al. (2012) focused on growth, which increased with Mg addition, but also Mg content and amino acid mobility in tea plants. Jayaganesh and Venkatesan, (2010) and Jayaganesh et al. (2011) also noted increased amino acid content with the addition of Mg, and Jayaganesh et al. (2011) observed that different forms of Mg had differential effects on catechin and carotenoid content of tea leaves. Increasing the supply of available N in soil was found to increased amino acid content of tea leaves (Venkatesan et al., 2005; Ruan et al., 2007b, 2010), although other studies (Han et al., 2008; Hamid et al., 2014) noted primarily increased growth with increasing N availability, rather than changes in plant composition.

### Carbon Dioxide

While this review did not retrieve any studies on the effects of carbon dioxide on tea quality, we summarize one recent study outside of the time frame of our review. Li et al. (2017) found that there was a significant increase in concentrations of total catechins and other polyphenols along with theanine and free amino acids with elevated carbon dioxide. Concurrently, levels of caffeine decreased (Li et al., 2017). These chemical findings are in sync with expression levels of biosynthetic genes for catechins and theanine that were up-regulated in tea leaves under elevated carbon dioxide levels while levels of biosynthetic genes for caffeine were down-regulated.

## Discussion and Conclusion

While the majority of research examining the effects of climate change on food systems has focused on crop yields (Lobell and Asner, 2003; Hertel et al., 2010; Lobell et al., 2011), this systematic literature review highlights that multiple environmental variables linked to climate change also significantly impact crop quality as measured by secondary metabolites associated with flavor attributes and health claims for human consumers (Wolfe et al., 2008; Liu, 2013). Specifically, findings provide evidence that shifts in seasonality, water stress, geography, light factors, altitude, herbivory and microbes, temperature, and soil factors that are linked to climate change can result in both increases and decreases up to 50% in secondary metabolites. A gap was found regarding evidence on the direct effects of carbon dioxide on tea quality, highlighting a critical research area for future study. The reviewed studies provides evidence that environmental factors linked to climate change can result in changes in the following biochemical measures of tea quality: phenolic catechins (i.e., epigallocatechin gallate, epicatechin gallate, epigallocatechin, and catechin gallate), total phenolic concentration, antioxidant activity, caffeine, theaflavin, thearubigin, tannins, carotenoids, terpene alcohols (i.e., linalool and geraniol) and other aromatic volatiles, fatty acids, chlorophyll, enzymes, and enzymatic activities. It is important to note that while an increase in many secondary metabolites represents an increase in crop quality, it may represent a decrease in crop quality for other secondary metabolites including those with off flavors or whose increased consumption by human consumers may result in toxicity (Kfoury et al., 2018). While this systematic review provides evidence that multiple environmental parameters are impacting tea quality, the directionality and magnitude of these impacts is not clear with contradictory evidence between studies. Findings build on a recent literature review highlighting the importance of understanding climate change effects on tea production (Marx et al., 2017) by providing evidence on the multi-directionality of changes in crop quality in response to shifts in environmental factors that are shifting with global change. As crop quality is a key determinant of health in the food system, findings have broader implications for other culturally-relevant crops managed and used by people for food, medicinal, and aesthetic purposes including fruits, vegetables, tree nuts, and beverage crops (Ahmed and Stepp, 2016).

The environmental factors with the most consistent evidence in this systematic review were seasonality and water stress. Specifically, a total of 14 out of 18 studies (78%) demonstrated a decrease in concentrations of phenolic compounds or their bioactivity with a seasonal shift from the spring and /or first tea harvest to other seasons. Seven out of ten studies (70%) showed an increase in levels of phenolic compounds or their bioactivity with drought stress. However, for both seasonality and water stress, multiple studies highlighted that while some secondary metabolite compounds increase, other compounds decrease, pointing to the complexity of understanding tea quality. Based on the evidence regarding seasonality and geographic variability highlighted in this study, tea quality is likely to increase during some parts of the year in some geographic areas with climate change (i.e., during periods of drought) while decreasing at other times of the year (i.e., during extended monsoon periods). However, if drought stress exceeds moisture thresholds of tea plants in a warming world, productivity will be jeopardized.

Herbivory and soil fertility were two of the variables that showed the greatest contradictory evidence with mixed impacts on tea quality. A surprising finding was that while many crop species are inhibited by high levels of soluble aluminum (Al) in the soil, we found multiple studies that demonstrated that Al not only stimulated tea growth (Mukhopadyay et al., 2012), but also phytochemical concentration associated with high-quality tea (Chen et al., 2011; Duan et al., 2012; Sae-Lee et al., 2012; Hajiboland et al., 2013). Both herbivory and soil fertility are variables which farmers have the greatest control over, pointing to the importance of agricultural management for climate mitigation and adaptation. Farmers can respond at some capacity to climate-driven shifts on crop quality at the level of individual fields or farms. However, beyond specific thresholds, adaptation would require geographic shifts in production, yet these thresholds are largely unknown for crop quality.

The contradictory evidence found in this systematic review likely results from multiple confounding factors associated with each study including variation in tea variety, cultivar, specific environmental and agricultural management conditions, and differences in methods that make comparisons across studies difficult. For example, previous research indicates that different varieties and cultivars of a specific species such as tea may respond variably to the same environmental factor such as seasonality (Owuor and Chavanji, 1986). Owuor and Chavanji (1986) found that seasonal fluctuations variably impacted the caffeine content of tea plants depending on the type of clone. Controlled experiments are called for that examine the impacts of specific environmental factors on multiple tea varieties and cultivars including climate resilient cultivars. In addition, future research efforts should focus on not just understanding the implications of individual factors on specific attributes of crops, but on modeling the system-wide impacts of simultaneous changes on crop biological systems to better understand the long-term implications of climate change. This includes understanding complex interactions of crops with the environment including dynamic feedback loops in which the presence and concentrations of secondary metabolites are impacted by a cascade of abiotic and biotic conditions such as changes in precipitation, temperature, and pests (Fields and Orians, 2006; Schepp, 2009). Research is further needed to analyze the quality of studies included in this systematic review and similar reviews regarding the effects of climate-related changes on crop quality.

The overall lack of consistency in evidence for climate-driven effects on tea quality, coupled with our inability to quantitatively compare study outcomes because of a lack of comparable measures and information on tea variety, points to the challenge of measuring crop quality and the multidimensional nature and complexity of this parameter. One way to overcome this challenge is to design more widely agreed upon international standards of crop quality for different culturally and economically relevant crops along with implementing global collaborative projects with shared experimental designs to allow for comparison of outcomes across multiple studies on crop quality through space and time.

In addition, the lack of studies examining the interplay of multiple environmental factors on tea quality highlights the need for an ecosystems approach in examining multi-trophic interactions in tea systems as well as implications for consumers and farmer livelihoods. Further research is needed to analyze the quality of studies included in this systematic review. While our synthesis contributes to elucidating the directionality of changes in tea quality in response to environmental change, we were unable to quantitatively compare study outcomes because of a lack of comparable measures. Future work to develop standardized protocols for measuring crop quality to enable monitoring of this parameter and comparisons between studies is also needed.

Multiple agricultural, physiological, and molecular innovations have been identified toward the development of climate resilient tea systems (Ahmed, 2018). At the agricultural level, climate mitigation strategies for tea farms include agricultural diversification, tree planting and maintaining vegetative cover, management of soil organic matter and carbon sequestration, water management, controlling pests and disease, and migration and relocating agroecosystems to more suitable locations (Ahmed and Stepp, 2016; Ahmed, 2018). Physiological and molecular innovations for enhancing climate resilience of tea farms include: (1) cultivation of tea from seed versus from clonal propagules, (2) micropropagation, (3) traditional breeding methods of climate-smart cultivars, (4) genetically modified crops and, (5) sustainable intensification (Ahmed, 2018). Traditional breeding methods of climate-smart tea cultivars including those with enhanced resistance to climate variability such as extreme drought, early- maturing varieties, and biofortified crops that accumulate more minerals and vitamins (Ahmed, 2018). Research is needed to measure the effectiveness of these strategies for enhancing the resilience of shifts in crop quality to climate change. While farmers can respond at some capacity to climate-driven shifts on crop quality at the level of individual fields or farms, changes beyond specific ecological and physiological thresholds would require geographic shifts in production. Future research is called for to elucidate what these thresholds are for crop quality for a range of staple and nutrient-dense crops that support food security and human health.

These findings have notable implications for the sustainability and resilience of agricultural systems in the context of global environmental change. Thus, it is essential to develop and implement evidence-based adaptation strategies for growing crops in ways that are resilient to climate change and support both environmental and human well-being. Based on our reflections on lessons learnt from this systematic review, we recommend the following next steps for advancing the understanding of climate change effects on crop quality as well as for responding to this challenge:

1. Carry out farm-level research on the effectiveness of various agricultural practices for climate adaptation and mitigation for tea plants and other food and beverage crops including the cost-effectiveness, replicability, and adaptability for different geographic contexts as well as differing scales and models of production. Findings should be applied to design and disseminate climate resilient agricultural guidelines that should be supported by policy including providing economic and social incentives.
2. Invest in breeding of climate-resilient crop cultivars that includes the evaluation of crop quality to multiple environmental factors and identification of crop quality thresholds.
3. Design more widely agreed upon international standards of crop quality for multiple culturally and economically relevant crops
4. Implement long-term global collaborative projects with shared experimental designs and “big data” sharing to allow for comparison of outcomes across multiple studies on crop quality through space and time. These studies should model system-wide impacts of simultaneous environmental changes on agricultural systems and implications for nutrition and human health to better understand the dynamic feedback loops in the food systems and associated social and ecological long-term implications of climate change.
5. Foster cross-sector collaboration between researchers, practitioners, producers, and policy makers to develop evidence-based adaptation strategies that reduce vulnerability of food systems to shifts in crop quality toward supporting sustainability.

## Data Availability

All datasets generated for this study are included in the manuscript and/or the Supplementary Files.

## Author Contributions

SA and TG conceived of the study question for the systematic review and guided the systematic review team. SA, TG, and MS designed the systematic review protocol with guidance from RH. All authors contributed to search terms. MS, SA, and MH served on the review panel to identify articles to be included in the study. DK, SA, DS, and AL served on the data extraction panel. SA, CM, and DK created the Figures and Tables. SA and TG wrote the manuscript with contributions from all authors. All authors approved the final manuscript submitted. SA and SC led the submission of the manuscript.

### Conflict of Interest Statement

The authors declare that the research was conducted in the absence of any commercial or financial relationships that could be construed as a potential conflict of interest.
