# Supplementary material for: Environmental Factors Variably Impact Tea Secondary Metabolites in the Context of Climate Change
Source: Front Plant Sci. 2019 Aug 13;10:939. doi: 10.3389/fpls.2019.00939 (PMC6702324; doi:10.3389/fpls.2019.00939)
Supplement: Supplementary file 1 [file Table_1.docx]

Supplementary Material

**Supplementary Table 1.** **Systematic review search terms.** This table lists the terms used in the systematic review literature search to answer the question: *What are effects of environmental variation related to climate change on tea quality?*
