# Supplementary material for: Environmental Factors Variably Impact Tea Secondary Metabolites in the Context of Climate Change
Source: Front Plant Sci. 2019 Aug 13;10:939. doi: 10.3389/fpls.2019.00939 (PMC6702324; doi:10.3389/fpls.2019.00939)
Supplement: Supplementary file 3 [file Table_3.docx]

Supplementary Material

**Supplementary Table 3. Effects of water stress on tea quality**
