# Supplementary material for: Environmental Factors Variably Impact Tea Secondary Metabolites in the Context of Climate Change
Source: Front Plant Sci. 2019 Aug 13;10:939. doi: 10.3389/fpls.2019.00939 (PMC6702324; doi:10.3389/fpls.2019.00939)
Supplement: Supplementary file 10 [file Image_1.pdf]

Supplementary Material

1 Supplementary Figure

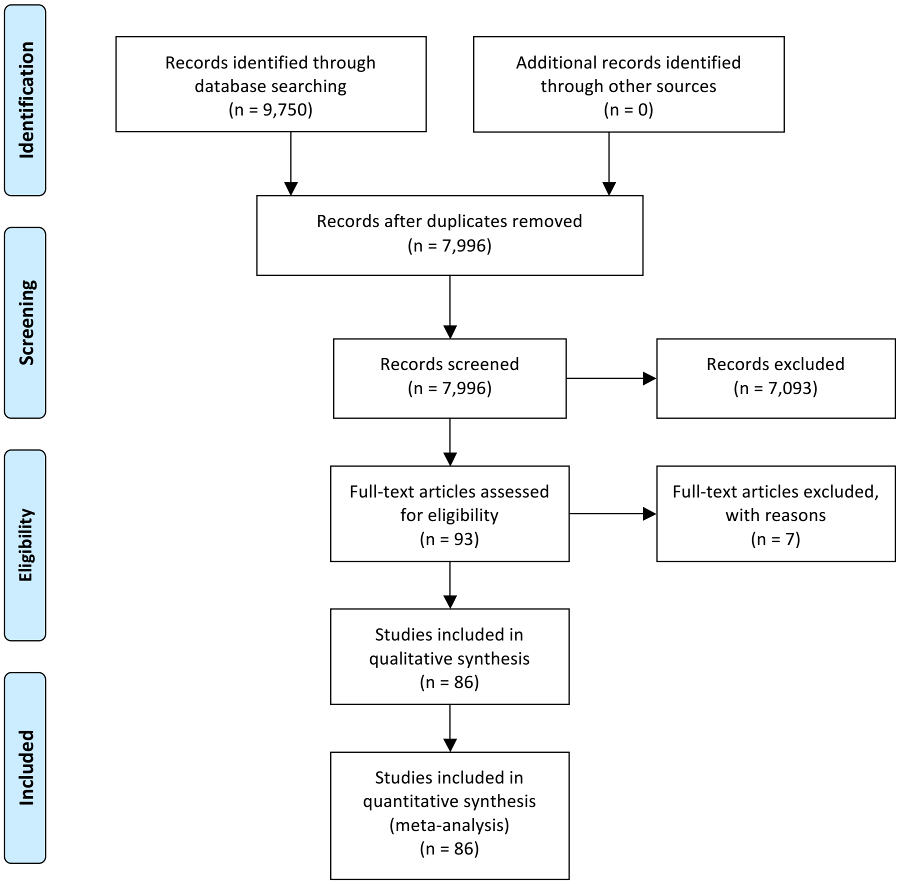

Supplementary Figure 1. PRISMA flow diagram. PRISMA flow diagram depicting the number of articles identified, screened, assessed for eligibility, and included in the systematic review synthesis.
